# First person – Maryam Hekmatara

**DOI:** 10.1242/bio.062363

**Published:** 2025-11-25

## Abstract

First Person is a series of interviews with the first authors of a selection of papers published in Biology Open, helping researchers promote themselves alongside their papers. Maryam Hekmatara is first author on ‘ Super-resolution microscopy reveals a Rab6a-dependent trafficking hub for rhodopsin at the mammalian rod photoreceptor Golgi’, published in BIO. Maryam is a postdoc in the lab of Dr Michael Robichaux at West Virginia University School of Medicine, Morgantown, WV, USA, investigating retinal cell biology and protein trafficking using super-resolution microscopy.


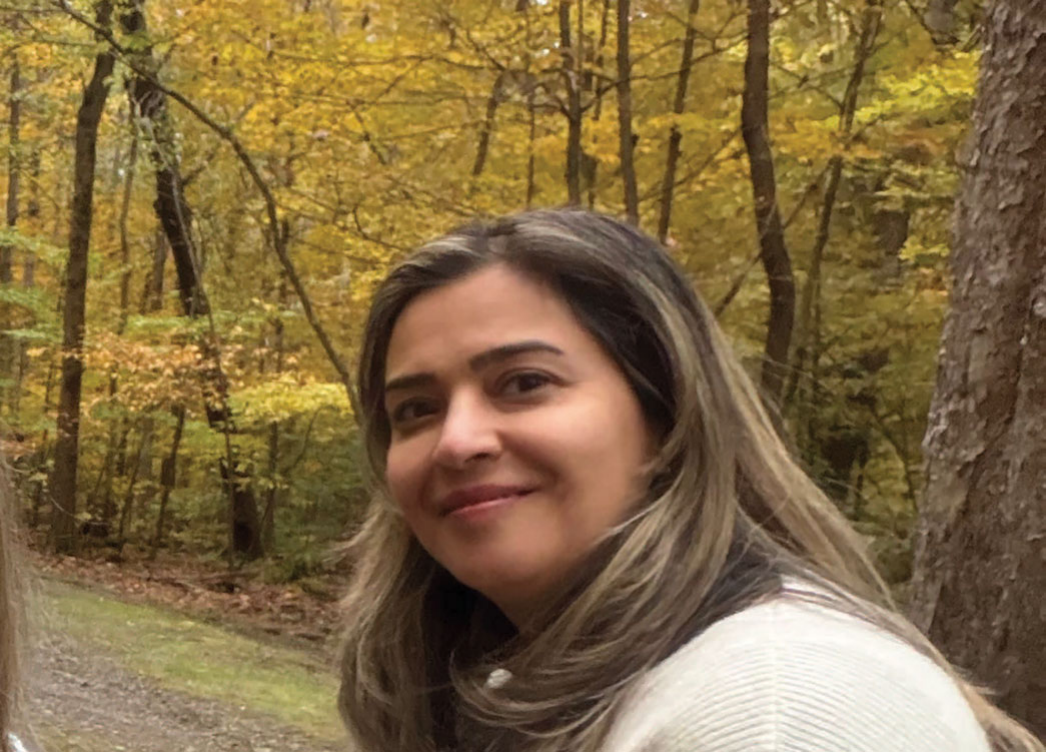


**Maryam Hekmatara**

**Describe your scientific journey and your current research focus**

My scientific journey began with a deep enthusiasm for elementary school science books, eventually leading me to pursue a BSc and MSc in life sciences nearly 1000 miles from my hometown. During my graduate studies, I discovered five new crustacean species and one new genus from Iranian inland waters and the southern coast along the Gulf of Oman. My passion for understanding life at the molecular level then led me to China, where I was awarded a full scholarship from Chinese Academy of Sciences and earned a PhD in biochemistry and molecular biology. There, I developed a novel platform combining Raman micro-spectroscopy with stable isotope probing to study cellular metabolic dynamics during drug–cell interactions. Continuing my global scientific path, I moved to the United States for a postdoctoral fellowship in vision neuroscience. My current research focuses on retinal cell biology and the mechanisms underlying retinal degeneration. I am especially interested in how cellular trafficking and protein misfolding contribute to vision loss, aiming to identify therapeutic targets for retinal diseases.

**Who or what inspired you to become a scientist?**

That's a question I often ask myself – how did I fall in love with science at the age of 8 or 9? From that young age, I knew I wanted to become a scientist, and that goal has never changed. One of my earliest memories is watching wildlife documentaries with my father, which I believe sparked my fascination with the natural world. Those moments lit a curiosity in me that only grew stronger over time. My parents also played a big role; they were often amazed by my questions and enthusiasm for science. Their encouragement and unwavering support for my education helped solidify my path. Looking back, it was a combination of wonder, curiosity and a nurturing environment that inspired me to pursue a life in science.

**How would you explain the main finding of your paper?**

Rhodopsin is a protein in the eye that helps us detect light, and, without it, we cannot see. For vision to work properly, rhodopsin has to be delivered to the correct place inside the light-sensing cells of the eye. If it ends up in the wrong place, these cells become damaged and eventually die, leading to vision loss. In our study, we showed that a part of the cell called the Golgi system acts like a sorting and shipping center that makes sure rhodopsin is delivered correctly. We found that when this system is disrupted, rhodopsin does not reach the right location, which helps explain how some forms of blindness begin.

… when [the Golgi] system is disrupted, rhodopsin does not reach the right location, which helps explain how some forms of blindness begin

**What are the potential implications of this finding for your field of research?**

This finding clarifies a fundamental step in rhodopsin biosynthetic trafficking within rod photoreceptors. By demonstrating that the cis- and trans-Golgi domains form distinct yet tightly aligned subcompartments critical for proper rhodopsin sorting, we identify the Golgi as a key regulatory node in maintaining photoreceptor polarity. This provides a mechanistic explanation for how disruptions in Golgi structure or function can lead to rhodopsin mislocalization and photoreceptor stress. These insights suggest that stabilizing Golgi organization or improving trafficking fidelity could represent new therapeutic strategies for rhodopsin-associated retinal diseases. Additionally, this work contributes to a broader understanding of how highly polarized neurons maintain protein distribution and functional compartmentalization.

**Figure:**
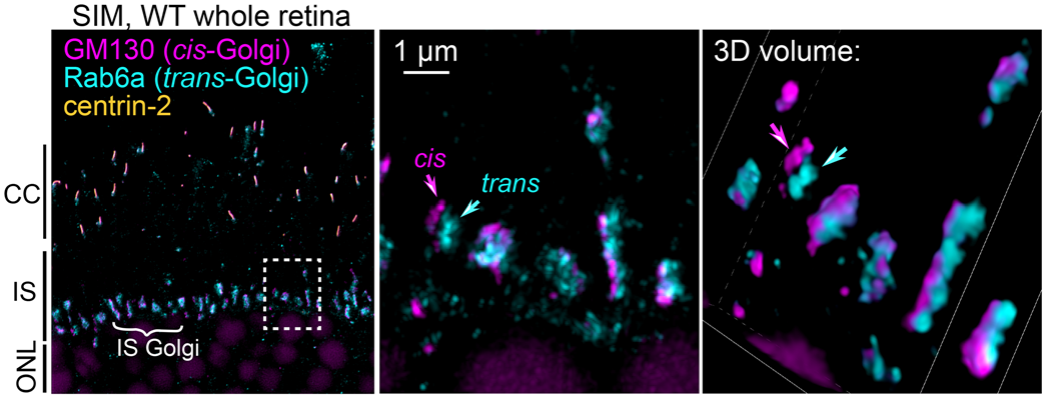
**An image of a wild-type (WT) mouse retina captured by SIM.** The 3D view demonstrates that Rab6a trans-Golgi can be identified as distinct Golgi fluorescent puncta (cyan arrows) from the GM130-labeled cis-Golgi (magenta arrows). CC, connecting cilia; IS, inner segment; ONL, outer nuclear layer.

**Which part of this research project was the most rewarding?**

When I visualized, using high-magnification structured illumination microscopy (SIM) imaging, that the cis-Golgi and trans-Golgi in individual rod photoreceptors form closely apposed yet completely non-overlapping subdomains. Seeing this precise spatial segregation at the sub-cellular level was a moment where the biology became visible – complex cellular architecture revealed in a way that felt both elegant and meaningful.

**What do you enjoy most about being an early-career researcher?**

What I enjoy most about being an early-career researcher is the feeling that my curiosity is still my compass. Coming from a background where I moved across countries and scientific fields, I value the freedom to ask new questions and learn continuously. Every day in the lab feels like a continuation of the same curiosity I had as a child wondering how life works. I also appreciate the opportunity to collaborate with people from diverse cultures and perspectives, which reflects my own international scientific journey. At this stage, nothing feels fixed – there is space to grow, to make mistakes, to be creative, and to shape the kind of scientist I want to become. That openness and sense of possibility is what excites me most.

**What piece of advice would you give to the next generation of researchers?**

Science can be difficult, unpredictable and slow, but that is also what makes it meaningful. Be patient with yourself, stay curious, and allow failure to be part of the process rather than a sign to stop. Seek out mentors and collaborators who lift you up, and be the person who does the same for others. Science grows through shared curiosity and courage. Keep going, stay open and trust that your journey has value.

**What's next for you?**

The next step in my career is to continue building an independent research path focused on understanding and treating neurodegeneration. I aim to deepen my work on retinal degeneration and protein trafficking in photoreceptors and to explore therapeutic strategies that can preserve or restore vision. I am currently seeking opportunities to further develop my expertise, strengthen collaborations, and contribute to translational research that can move discoveries closer to patients. In the long term, I hope to establish my own lab where I can mentor future scientists and continue this line of investigation.

**What changes do you hope to see in the academic environment for early-career researchers, particularly postdocs?**

Postdocs are a vital part of the scientific workforce, yet they often face job insecurity, limited funding stability, and a lack of clear long-term career pathways. Despite being highly trained and deeply committed to advancing research, many are forced to make significant personal and professional sacrifices without the assurance of a sustainable future in academia. Strengthening institutional support for postdocs – through more stable positions, clearer mentoring structures and transparent pathways to independence – would not only improve postdoc well-being, but also help preserve the next generation of scientific leaders. Investing in postdocs means investing directly in the future of research, discovery and innovation. To maintain a thriving academic landscape, we must create conditions where talented researchers can build secure and fulfilling careers, rather than be pushed away by uncertainty.
